# Reply to Sanfilippo et al. Caution Is Warranted When Assessing Diastolic Function Using Transesophageal Echocardiography. Comment on “Kyle et al. Consensus Defined Diastolic Dysfunction and Cardiac Postoperative Morbidity Score: A Prospective Observational Study. *J. Clin. Med.* 2021, *10*, 5198”

**DOI:** 10.3390/jcm11123300

**Published:** 2022-06-09

**Authors:** Mateusz Zawadka, Bonnie Kyle, Hilary Shanahan, Jackie Cooper, Andrew Rogers, Ashraf Hamarneh, Vivek Sivaraman, Sibtain Anwar, Andrew Smith

**Affiliations:** 1 Perioperative Medicine, Barts Heart Centre, St. Bartholomew’s Hospital, London EC1A 7BE, UK; bonnie.kyle@nhs.net (B.K.); dr.amrogers@gmail.com (A.R.); a.hamarneh@ucl.ac.uk (A.H.); m.t.zawadka@gmail.com (V.S.); s.anwar@qmul.ac.uk (S.A.); andrew.smith84@nhs.net (A.S.); 2 NIHR Biomedical Research Centre, William Harvey Research Institute, Barts, Queen Mary University of London, London E1 4NS, UK; jackie.cooper@qmul.ac.uk; 3 2nd Department of Anesthesiology and Intensive Care, Medical University of Warsaw, 02-091 Warsaw, Poland; 4 Department of Anaesthesia and Critical Care, Papworth Hospital NHS Foundation Trust, Papworth Everard, Cambridge CB2 0AY, UK; hshanahan@nhs.net; 5 Outcomes Research Consortium, Cleveland Clinic, Cleveland, OH 44195, USA

We thank Sanfilippo and colleagues for their insightful comments about the assessment of diastolic function with transesophageal echocardiography (TEE) [1]. In our investigation [2] we used the current guidelines for assessment of diastolic function by the American Society of Echocardiography (ASE) and the European Association of Cardiovascular Imaging [3]. Although yet to be formally validated for the assessment of left ventricular diastolic dysfunction (LVDD), TEE continues to be used in clinical practice both in the operating theatre and ICU. The ASE recommend TEE for evaluation of LVDD as part of an ongoing intraoperative study [4,5], accepting the fact that this may be prone to unavoidable inaccuracy [6].

Of note, we outlined our process of diagnosis and grading of LVDD in “Appendix 3: Process of Evaluating DD”. Within those known limitations, our results showed that trying to follow the guideline allowed characterisation of most of the studied patients. Regarding the issues raised against the use of left atrial volume index (LAVI) and tissue Doppler imaging (TDI), we take this opportunity to further clarify the process.

Firstly, 30% of patients had depressed ejection fraction and, thus, a diagnosis of diastolic dysfunction existed by definition, independently from intraoperative assessment. Secondly, we agree with Sanfilippo and colleagues that the LAVI measurement is flawed in TEE due to the anatomical difficultly of fully visualizing the left atrium (LA). However, in most patients, satisfactory images of the LA were obtained (*n* = 107) and then used to calculate the LAVI. Moreover, even if the diagnosis of mildly increased LAVI can be challenging with TEE, it is relatively easy to identify a severely dilated LA and to use this in the assessment of LVDD [5]. As clinicians, we have to accept the flaws of the measurements and be aware of the limitations.

Thirdly, considering the results of the study by Mauermann [7] and colleagues, we are aware of the possible underestimation of relaxation (e’), but as suggested by the authors, this difference is modest and probably not clinically relevant, being in the region of 0.6 cm/s. Therefore, the impact on LVDD is unremarkable from a pragmatic perspective. 

A different aspect suggested by Sanfilippo and colleagues was the adherence to the PRICES guidelines for reporting critical-care echocardiography studies [8]. Although we agree that following these guidelines could strengthen the study and make it more comparable with future studies, it is also true that the PRICES project was preceded by a systematic appraisal of the literature [9], which excluded the cardiac surgery setting. Although prospective, our study enrolled patients undergoing cardiac surgery from November 2014 to December 2016, years before the PRICES recommendations were issued. For these reasons we did not collect and thus report several data deemed essential by the PRICES recommendations. 

When conducting ultrasound research, it is also vitally important to acknowledge variability in different learning pathways and ensure standardisation of competences [10,11,12,13,14]. Critical-care echocardiography research, due to its limitations (critically ill patients, time sensitive exams and patients’ heterogeneity), tends to be low volume and a comparable reporting system would further strengthen the field and provide more robust findings. Research in an intensive care setting can be especially challenging as most of the available guidelines refer to the general public and its application to different populations might be controversial. There is a need for a collaborative and multicentered effort in critical-care echocardiography to provide enough high-quality evidence to build guidelines for this very specific population.
